# Fetuin-A Promotes 3-Dimensional Growth in LNCaP Prostate Cancer Cells by Sequestering Extracellular Vesicles to Their Surfaces to Act as Signaling Platforms

**DOI:** 10.3390/ijms23074031

**Published:** 2022-04-05

**Authors:** Josiah Ochieng, Olga Y. Korolkova, Guoliang Li, Renjie Jin, Zhenbang Chen, Robert J. Matusik, Samuel Adunyah, Amos M. Sakwe, Olugbemiga Ogunkua

**Affiliations:** 1 Department of Biochemistry, Cancer Biology, Neuroscience and Pharmacology, Meharry Medical College, Nashville, TN 37208, USA; okorolkova@mmc.edu (O.Y.K.); gli@mmc.edu (G.L.); zchen@mmc.edu (Z.C.); sadunyah@mmc.edu (S.A.); asakwe@mmc.edu (A.M.S.); oogunkua@mmc.edu (O.O.); 2 Department of Urology and Vanderbilt –Ingram Cancer Center, Vanderbilt University Medical Center, Nashville, TN 37209, USA; renjie.jin@vumc.org (R.J.); robert.matusik@vumc.org (R.J.M.)

**Keywords:** fetuin-A, vesicles, exosomes, 3-dimensional, 2-dimensional, growth, anchorage, signaling, prostate, cancer

## Abstract

The present studies were conducted to evaluate key serum proteins and other components that mediate anchorage-independent growth (3-D growth) of LNCaP prostate cancer cells as spheroids. The cells were cultured on ultra-low attachment plates in the absence and presence of fetuin-A and with or without extracellular vesicles. The data show that fetuin-A (alpha 2HS glycoprotein) is the serum protein that mediates 3-D growth in these cells. It does so by sequestering extracellular vesicles of various sizes on the surfaces of rounded cells that grow as spheroids. These vesicles in turn transmit growth signals such as the activation of AKT and MAP kinases in a pattern that differs from the activation of these key growth signaling pathways in adherent and spread cells growing in 2-D. In the process of orchestrating the movement and disposition of extracellular vesicles on these cells, fetuin-A is readily internalized in adhered and spread cells but remains on the surfaces of non-adherent cells. Taken together, our studies suggest the presence of distinct signaling domains or scaffolding platforms on the surfaces of prostate tumor cells growing in 3-D compared to 2-D.

## 1. Introduction

We previously reported that fetuin-A mediates the biogenesis of extracellular vesicles that mediate the attachment of tumor cells to various extracellular matrices [1]. Fetuin-A is a multifunctional extracellular protein that data suggest is a hub gene that intersects with a number of pathways critical for tumor growth and metastasis [2,3]. It is mostly synthesized by the liver and other organs and secreted into the blood [4]. Whereas its concentration in human fetal blood can be as high as 2 mg/mL, the concentration in adult blood is approximately 0.6 mg/mL [5]. Recently, a couple of studies demonstrated that it can also be synthesized ectopically by some tumor cells and tissues [6,7]. Published reports regarding the interactions of fetuin-A with tumor cells come from two-dimensional (2-D) growth studies, which show that fetuin-A is rapidly internalized by attached and spread cells [8,9]. This rapid uptake of fetuin-A is a critical step that drives the rapid spreading and attachment of the tumor cells to the substrata. Consequently, inhibition of this rapid uptake attenuates the rapid attachment and spreading of the cells [9]. Cellular attachment and spreading then initiate growth signaling via attachment receptors, including integrins. The data suggest that fetuin-A mediates the uptake of extracellular vesicles and, in particular, exosomes that in turn transmit rapid attachment and spreading signals to the recipient cells [1,10]. Exosomes synthesized and secreted by tumor cells in the absence of fetuin-A lack the ability to facilitate the rapid spreading of cells [1]. 

It is becoming clearer that the 3-dimensional (3-D) growth of tumor cells, also known as anchorage-independent growth (AIG), is mediated by growth factors and a plethora of extracellular vesicles (EVs) such as exosomes [11,12] and small and large extracellular vesicles [13]. However, the precise molecular mechanisms by which these EVs collaborate with growth factors to orchestrate the 3-D growth of tumors are not clear. In the present studies, we have investigated the mechanisms by which fetuin-A modulates the 3-D growth of tumor cells using LNCaP prostate cancer cells as a model system. LNCaP cells are normally propagated in a cell culture medium containing 10% fetal bovine serum (FBS) in high attachment culture plates, where they adhere and grow in an anchorage-dependent fashion. However, they can also grow rapidly as spheroids (anchorage-independent growth) in a culture medium containing 10% FBS when cultured on low or ultra-low cell attachment culture plates [14]. Obviously, FBS, used to supplement the growth medium, has a host of growth factors, including androgens, that are key factors for prostate cancer cells, particularly LNCaP [15] and exosomes that promote AIG [16,17]. FBS and human sera have high concentrations of fetuin-A, which was recently demonstrated to be a biomarker for prostate cancer metastasis [6].

In so far as prostate tumor growth is concerned, the majority of tumors require androgens for their optimal growth, and androgen deprivation therapy is one of the standard treatment modalities for prostate cancer [18]. However, there are more aggressive phenotypes of prostate cancer that no longer require androgens, termed castration-resistant prostate cancer (CRPC) [19]. It is, therefore, reasonable to speculate that CRPC cells have acquired other growth-promoting mechanisms that override their requirements for androgens. Such mechanisms include androgen receptor variants that are constitutively activated [20]. Other developments may involve the increased synthesis of fetuin-A by the tumor cells and/or the cell surface receptor for fetuin-A. A recent report by Mintz et al. [6] indicated increased expression of fetuin-A in prostatic tissues in patients who had castration-resistant prostate cancer, with the implication that fetuin-A was a driver in the progression of these tumor cells [6]. 

The present studies were conducted in order to delineate how fetuin-A modulates the 3-D growth of LNCaP prostate cancer cells as spheroids. Such growth mechanisms closely resemble the growth characteristics of tumor cells in vivo. We hereby show that fetuin-A, a serum glycoprotein has a causal effect on the 3-D growth of LNCaP prostate cancer cells.

## 2. Results

### 2.1. Fetuin-A Is Indispensable in the 3-D Growth of LNCaP Cells

**Figure 1:**
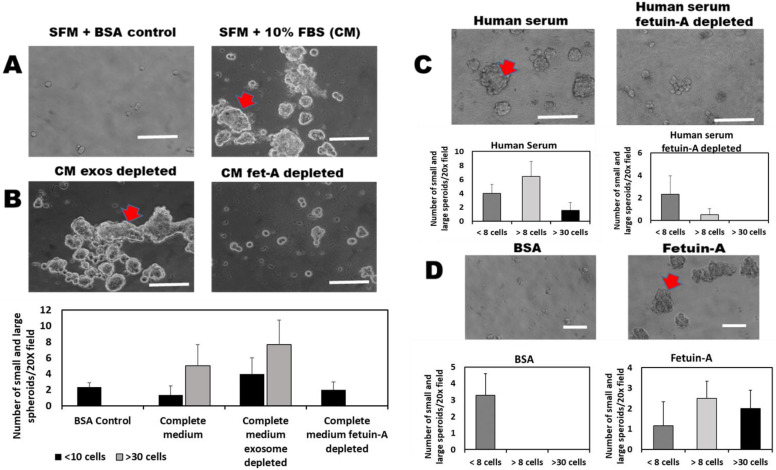
Fetuin-A is the serum factor that promotes 3-D growth of LNCaP cells. In panels (**A**,**B**), cells were dislodged with Trypsin/EDTA, counted, and then plated as single-cell suspensions in an ultra-low 24-well attachment (1 × 10^4^ cells/well) in SFM+BSA (negative control); SFM+10% FBS (CM); CM depleted of exosomes; and CM depleted of fetuin-A. The number of spheroid colonies of different sizes was counted (20× microscopic field) after 10 days of growth, as depicted. In panel (**C**), single cells (1 × 10^4^ cells/well) were plated in SFM+10% adult human serum (Human serum) or SFM+adult human serum depleted of fetuin-A. The number of the different sizes of spheroid colonies in a 20× field (N = 6) was determined after 10 days of growth. In panel (**D**), single cells again were plated as above in SFM+BSA (negative control) or SFM+purified fetuin-A. The numbers of the different sizes of spheroid colonies in a 20× field are shown. Scale bars in panels (**A**–**C**) = 100 µm; in panel (**D**) = 50 µm. The growth assays were repeated 5 times.

We hereby showed that complete medium (CM) containing 10% fetal bovine serum (FBS) supports a robust 3-D spheroid growth of LNCaP cells in low or ultra-low attachment plates, as expected, while serum-free medium (SFM) containing bovine serum albumin (BSA) (2 mg/mL) (control) does not (Figure 1A). Spheroids with more than 30 cells (large spheroids; red arrowheads), as well as medium-sized spheroids (>8 cells but less than 30 cells), were only observed in wells containing complete medium. Control wells containing BSA only had small spheroids that had less than eight cells and represented a lack of 3-D growth. Only medium-sized and large-sized spheroids represented true 3-D growth.

We also demonstrated that the 3-D growth of these cells in CM is not due to the endogenous bovine serum exosomes in that CM depleted of these exosomes continue to robustly support the 3-D growth of LNCaP cells (Figure 1B, left panel). Interestingly, fetuin-A depleted CM failed to support the 3-D growth of the cells (Figure 1B, right panel). Only small spheroids with less than eight cells were observed in wells containing fetuin-A depleted CM. Similarly, serum-free medium containing 10% human serum supported the 3-D growth of LNCaP cells (Figure 1C, left panel), while human serum depleted of human fetuin-A (2HS glycoprotein) did not support 3-D growth of the tumor cells (Figure 1C, right panel). To demonstrate that it is fetuin-A alone that supports 3-D growth and not fetuin-A complexed with other serum proteins, we repeated the experiments using purified fetuin-A (2 mg/mL) or BSA (2 mg/mL) in SFM. It was clear that fetuin-A by itself supports 3-D growth of LNCaP cells (Figure 1D, right panel). There were several large-sized spheroids with more than 30 cells (red arrowhead) and a similar number of medium-sized spheroids within a 20× field of view. BSA control, on the other hand, did not support 3-D growth. No medium-sized nor large-sized spheroids were observed (Figure 1D, left panel).

### 2.2. 3-D Growth of LNCaP Cells Is Mediated by Exosomes and Other Extracellular Vesicles Isolated from the Cells in the Presence of Fetuin-A

**Figure 2:**
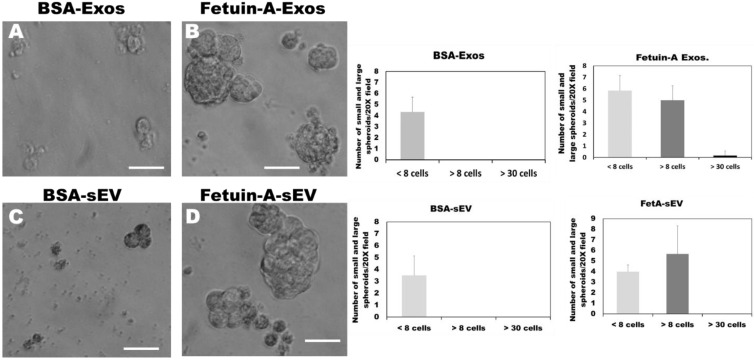
Extracellular vesicles isolated in the presence of fetuin-A mediate the 3-D growth of LNCaP cells. Extracellular vesicles (exosomes and ‘sEVs’) were isolated as described in Materials and Methods. Single cells (1 × 10^4^ cells/well) were plated in an ultra-low attachment 24-well plate in serum-free medium containing BSA-exosomes (50 µg/well) (panel **A**); Fetuin-A exosomes (50 µg/well) (panel **B**); BSA-sEV (50 µg/well) (panel **C**); and Fetuin-A-sEV (50 µg/well) (panel **D**). The number of the different colony sizes (per 20× field; N = 6) obtained under each condition was determined after 10 days of growth and graphed as shown. Scale bars = 50 µm. The experiment was repeated 5 times.

We next wanted to elucidate the molecular mechanism by which fetuin-A promotes the 3-D growth of LNCaP cells. We have established that fetuin-A modulates adhesion and cell spreading via exosomes secreted by the tumor cells [1,10,21]. Only exosomes isolated from the tumor cells in the presence of fetuin-A mediated cellular adhesion. We considered these exosomes as ‘bioactive’. Exosomes isolated in the absence of fetuin-A failed to promote cellular adhesion [1]. We, therefore, questioned whether the ‘bioactive’ exosomes could also modulate the 3-D growth of tumor cells. We isolated exosomes, large (LEV) and small (sEV), extracellular vesicles from 1 × 10^9^ LNCaP cells in large 150 cm^2^ flasks, as described. As with cellular adhesion and spreading, only exosomes isolated from LNCaP cells in the presence of fetuin-A were able to promote the 3-D growth of cells (Figure 2B). There were numerous medium-sized and a few large-sized spheroids. Exosomes isolated in the presence of BSA control failed to promote 3-D growth (Figure 2A). There were a few small-sized spheroids with less than eight cells. The sizes of the exosomes we isolated, 139 ± 70 nm, are comparable to those that have been reported by others [17]. As with the exosomes, only sEV (pelleted at 20,000× *g*) isolated in the presence of fetuin-A promoted 3-D growth of LNCaP cells (Figure 2D). There were numerous mid-sized spheroids with more than 8 cells but less than 30 cells (3-D growth) and almost an equal number of small-sized spheroid colonies (<8 cells) that were expanding in size. However, the number of large-sized spheroids (>30 cells) was very few, with most microscopic fields of view (20×) not showing any large-sized spheroid. The sEV isolated in the presence of BSA did not have any medium-sized or large-sized spheroids (Figure 2C). The sEV were slightly larger than exosomes, with a mean diameter of 208 nm ± 82. Similar data were obtained with LEVs, which were the largest of the extracellular vesicles (240 ± 117 nm).

### 2.3. Fetuin-A Sequesters Rhodamine Isothiocyanate Labeled Large Extracellular Vesicles (LEV) on the Surfaces of Cells Growing in 3-D but Internalizes These Vesicles in Adhered and Spread LNCaP Cells

**Figure 3:**
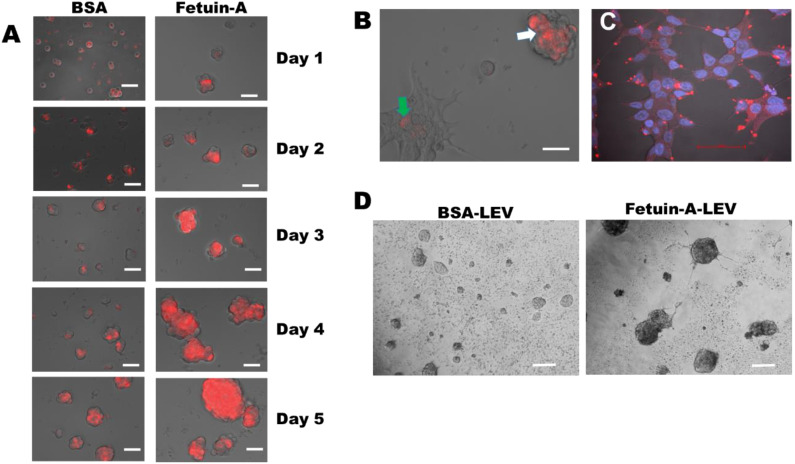
Fetuin-A drives the internalization of labeled LEV in adhered cells but sequesters them on the surfaces of spheroid cells or non-adhered cells. Large extracellular vesicles (LEV) were isolated from LNCaP cells in SFM containing BSA, as described in Materials and Methods. The vesicles were labeled with rhodamine isothiocyanate, purified by passing twice through the desalting column, and again by centrifugation twice (3000× *g*) for 15 min each. The pellet was resuspended in SFM to a concentration of 1 mg/mL. In panel (**A**), LNCaP cells were plated in 96-well low attachment plates (5000 cells/well) in SFM containing BSA or fetuin-A (2 mg/mL). To each well, 15 µg of labeled LEV was added, and the cells were incubated for 5 days. The cells with labeled LEV were photographed every day for 5 days under a Keyence epifluorescence microscope adjusted to acquire fluorescence images of cells growing on plastic plates. In (**B**), the same number of cells was plated on cell culture-treated high attachment 96-well plates. Each well contained 15 µg of labeled LEV. The wells were photographed after 4 days, by which time most of the label in the spread cells had dissipated. In (**C**), the cells were plated on glass coverslips in complete medium. After 24 h, labeled LEV (10 µg) was added to the adhered cells in complete medium and incubated for 2 h, fixed in 3.5% paraformaldehyde in PBS, washed, and then the coverslip was placed upside down on a drop of anti-fade with DAPI on a slide and images acquired by confocal microscopy (Nikon A1R). In (**D**), the cells growing in low attachment 96-well plates containing 15 µg/well of unlabeled LEV in SFM containing either BSA or fetuin-A (scale bars in panels (**A**–**D**) = 50 µm). The experiment was repeated 3 times.

In order to determine how fetuin-A influenced the disposition of LEV on either attached or rounded cells, we added rhodamine isothiocyanate labeled LEV (15 µg/well) to LNCaP cells in the wells of either ultra-low attachment (Figure 3A) or cell culture-treated (Figure 3B, left panel) 96-well microtiter plates (5000 cells/well). The cells growing in ultra-low attachment wells were allowed to grow for 5 days, and images were acquired daily under a Keyence microscope. 

Interestingly, labeled LEVs that were added to adhered and spread cells were quickly internalized within a 24-h period (green arrow), and only 3-D spheroid cells that failed to attach and spread had appreciable amounts of labeled LEV still remaining on their surfaces (white arrow), as shown in Figure 3B. To demonstrate that in attached and spread cells fetuin-A promotes the rapid uptake of rhodamine isothiocyanate labeled LEV, 2 h after adding labeled LEV in the presence of fetuin-A to attached and spread LNCaP cells, they were fixed with 3.5% paraformaldehyde in PBS, washed once with PBS, and then imaged on A1R confocal microscope. DAPI was added to the cells to visualize the nucleus prior to fixation. Within two hours, rhodamine isothiocyanate labeled LEVs were internalized and could be seen as large dots in the perinuclear regions (Figure 3C). Within 24-48 h, the internalized vesicles were metabolized, and the rhodamine isothiocyanate label dissipated. The sequestration of unlabeled LEVs by fetuin-A on the surfaces of LNCaP cells growing as spheroids was also evident under an inverted light microscope. LEVs were highly concentrated on the surfaces of rapidly growing spheroids of LNCaP cells in the presence of fetuin-A and appeared dark (Figure 3D, right panel) compared to the same concentration of LEVs added to the same number of cells in the presence of BSA as the control growing under similar conditions (Figure 3D, left panel). 

### 2.4. Large Extracellular Vesicles Associated with Labeled EGF Promote Rapid 3-D Growth of LNCaP Cells while Sequestered on the Cell Surface

**Figure 4:**
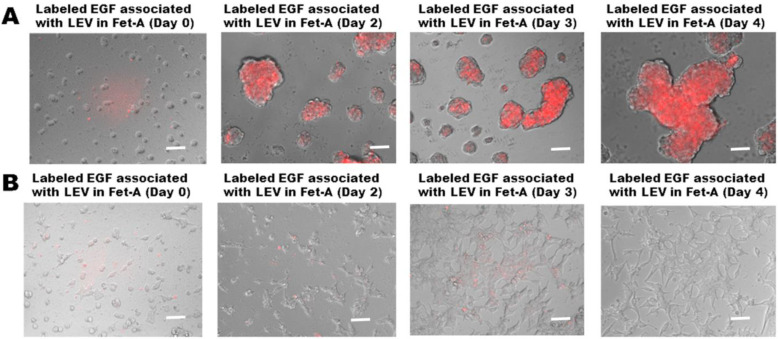
Large extracellular vesicles (LEV) associated with EGF remain sequestered on the cell surface to promote 3-D growth. LEVs were loaded with rhodamine isothiocyanate labeled EGF and then added (15 µg/well) to LNCaP cells (5000 cells/well) plated on either a low attachment plate (Panel (**A**)) or a cell culture-treated high attachment plate (Panel (**B**)) and then allowed to grow for four days. On days 2, 3, and 4, after plating the cells, the images of the spheroids or attached spread cells associated with labeled EGF were acquired by a Keyence epifluorescence microscope. Scale bar = 50 µm. The experiment was repeated 3 times.

In order to determine whether LEV loaded with EGF in the presence of fetuin-A would further enhance the 3-D growth of LNCaP cells, we performed 2-D and 3-D growth assays in the presence of fetuin-A and LEVs loaded with rhodamine isothiocyanate labeled EGF as the readout. As shown in Figure 4A, by day 2, there were already large spheroids with more than 30 cells. More importantly, the vesicles together with the associated labeled-EGF remained on the cell surface even as spheroids grew bigger, again suggesting that fetuin-A sequestered them on the cell surface. In comparison, the same number of cells to which the same concentration of LEV associated with labeled EGF was added but growing in 2-D only reached a confluency level of ~10% by day 2. The cells growing in 2-D rapidly internalized the EGF/LEVs and metabolized them such that after 4 days of growth, no labeled EGF was observed in LNCaP cells (Figure 4B). We repeated this experiment three times and obtained similar data.

### 2.5. Fetuin-A Is Rapidly Internalized by Attached and Adhered Cells while in Detached or Cells Growing as Spheroids, It Remains on the Cell Surface

**Figure 5:**
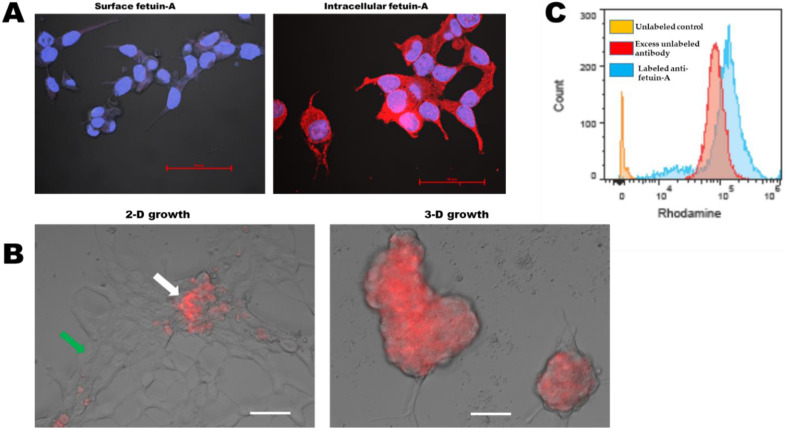
Fetuin-A is rapidly internalized by adhered cells, while in detached or spheroid cells, it remains on the cell surface. In (**A**), left panel, LNCaP cells were cultured on glass coverslips in the presence of fetuin-A (2 mg/mL) for 48 h. The cells were washed 3× in SFM and then fixed in 3.5% paraformaldehyde in PBS containing 1% BSA for 15 min. They were washed once with PBS and incubated with rhodamine isothiocyanate labeled rabbit polyclonal antibodies to fetuin-A. The cells were washed 5× with PBS containing 1% BSA, and the glass coverslips were turned upside down on a drop of anti-fade mounting solution with DAPI on microscope slides and images acquired by confocal A1R microscope (Nikon). In (**A**), right panel, LNCaP cells were cultured in SFM containing fetuin-A (2 mg/mL) and fixed in cold methanol for 5 min. They were incubated with rhodamine isothiocyanate labeled rabbit polyclonal antibodies to fetuin-A for 1 h at room temperature, washed, and processed for confocal microscopy (Scale bar = 50 µm). In (**B**), rhodamine isothiocyanate labeled fetuin-A (50 µg/well) was added to LNCaP that were grown on high attachment plates (2-D growth) or ultra-low attachment plates (3-D) for 4 days. The images were acquired by a Keyence epifluorescence microscope (scale bars = 50 µm). In (**C**), detached LNCaP cells were incubated in SFM containing fetuin-A (2 mg/mL) for 30 min at 37 °C. The cells were washed 5× with cold FACS buffer and fixed in paraformaldehyde. The fixed cells were incubated in cold FACS buffer (unlabeled controls; orange peak) or FACS buffer containing labeled polyclonal anti-fetuin-A (1:100 dilution; blue peak) or in buffer containing both labeled anti-fetuin-A (1:100) and excess unlabeled antibodies (1:5; red peak). The cells were washed 5× with FACS buffer and then analyzed by flow cytometer. The experiment was repeated 3 times.

Having demonstrated that fetuin-A is responsible for the sequestration of LEVs on the surfaces of LNCaP cells growing in 3-D as spheroids, we next questioned when fetuin-A itself is also concentrated or retained on the surfaces of these cells when they were spherical. To begin with, we determined that hardly any fetuin-A was detectable on the surfaces of intact cells that were adhered and spread. Rhodamine isothiocyanate labeled polyclonal antibodies against fetuin-A did not detect surface fetuin-A in cells that had been fixed with 3.5% paraformaldehyde (Figure 5A, left panel), while the same antibody easily detected the presence of intracellular fetuin-A that had been fixed in cold methanol where the membrane had holes that allowed antibody to freely enter the cells (Figure 5A, right panel). To detect fetuin-A on attached and detached spheroid cells, we added rhodamine isothiocyanate labeled fetuin-A to attached and spread cells as well as cells growing as spheroids on ultra-low attachment plates. As expected, labeled fetuin-A was rapidly internalized by attached and spread cells, indicated by the green arrow (Figure 5B, left panel). The internalized labeled protein was degraded, and the label dissipated. However, the labeled fetuin-A failed to enter a few cells that had not attached and spread, as shown by the white arrow (Figure 5B, left panel). Again, as expected, labeled fetuin-A remained on the surfaces of cells growing in 3-D as spheroids (Figure 5B, right panel). Lastly, we were able to observe the presence of fetuin-A on the surfaces of intact detached and spherical LNCaP cells when probed with rhodamine isothiocyanate-labeled polyclonal anti-fetuin-A antibodies by FACS (Figure 5C, blue peak). To determine the specificity of the antibody, we demonstrated that it could be quenched or competed in the presence of excess unlabeled anti-fetuin-A antibodies (Figure 5C, red peak). The orange peak represented cells incubated with unlabeled non-immune IgG (controls). 

### 2.6. Growth Signaling in LNCaP Cells

**Figure 6:**
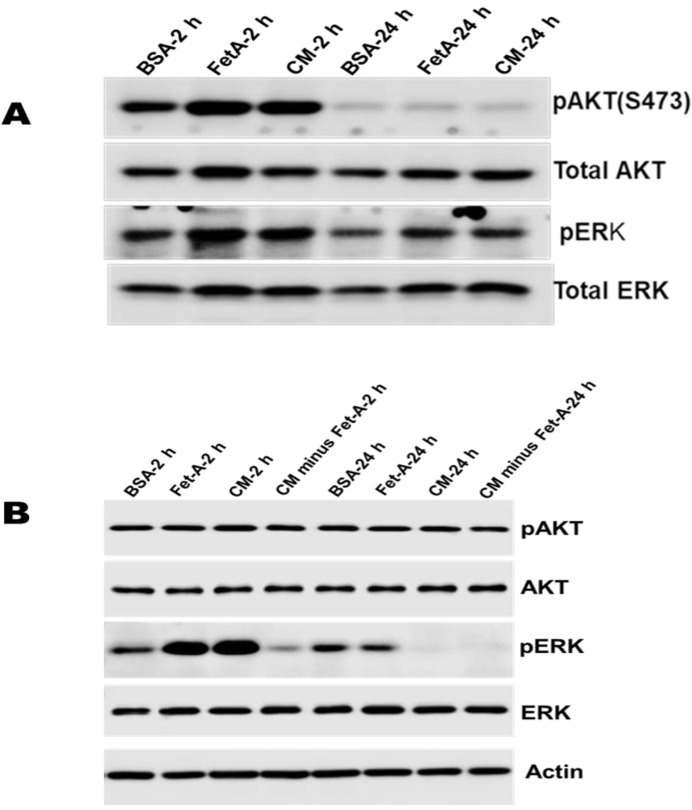
Distinct roles of fetuin-A on the activation of AKT and ERK in LNCaP cells growing in 2-D or 3-D. In (**A**), Cells growing in (2-D) culture flasks were serum-starved, and then the medium was changed to SFM containing BSA, Fetuin-A, or CM and incubated for the indicated time points, after which the cells were lysed in RIPA buffer and analyzed by Western blotting. As shown, activated AKT (pAKT) was slightly elevated in the presence of fetuin-A and CM compared to BSA, 2 h after serum starvation. However, after 24 h, pAKT had disappeared. Activated ERK, on the other hand, was evident for 24 h. In (**B**), the cells were grown in high attachment culture flasks until ~ 70% confluent. The cells were serum-starved, detached, and then re-plated in SFM containing BSA, fetuin-A, CM, and CM depleted (minus) of fetuin-A in ultra-low attachment flasks (3-D growth) and incubated for the indicated periods, after which the cells were lysed in RIPA buffer and analyzed by Western blotting. Of note was the high activation of ERK (pERK) in the presence of fetuin-A and CM, 2 h after serum starvation. Interestingly the activation was not observed in CM depleted of fetuin-A, with the implication that fetuin-A was responsible for ERK activation. After 24 h, ERK was still activated in the presence of fetuin-A but not CM. However, AKT was activated for the entire 24 h after serum starvation in the presence of BSA, fetuin-A, CM, as well as CM depleted of fetuin-A.

In order to determine how fetuin-A modulates growth signals in LNCaP cells, we compared the expression levels of activated forms of serine/threonine-protein kinase (AKT) and extracellular signal-regulated kinase (ERK) in cells growing in 2-D and 3-D over a 24-h period. In 2-dimensional growth, pAKT was elevated within 2 h following a 48 h of serum starvation in the presence of BSA, fetuin-A, or complete medium (CM). The levels of activated AKT were reduced to a low, almost undetectable baseline after 24 h in all three conditions, with total AKT used as a loading control. On the other hand, ERK was activated in the presence of BSA, fetuin-A, and CM within 2 h following the serum starvation and remained activated 24 h later, particularly in the presence of fetuin-A and CM (Figure 6A).

In 3-D growth, AKT remained activated over a 24 h period following serum starvation. Fetuin-A and CM elevated the levels of pERK considerably within 2 h following serum starvation compared to the BSA control. Interestingly, CM, which was denuded of fetuin-A, did not activate the MAP kinase compared to BSA control within the same period, suggesting that fetuin-A in CM was largely responsible for ERK activation. However, after 24 h, the pERK band was still visible in the presence of BSA and fetuin-A, but no activation was detected in the presence of CM and CM minus fetuin-A after 24 h (Figure 6B). 

## 3. Discussion

In the present studies, we provided experimental evidence that fetuin-A, a liver-derived glycoprotein, is the key serum factor that mediates 3-dimensional (3-D) growth of a prostate cancer cell line, LNCaP. We previously demonstrated that fetuin-A is a major driver of tumor cell motility and invasion of head and neck squamous cell carcinoma [22] and glioblastoma [7] cell lines in 2-dimensional (2-D) model systems. The impetus for the present studies was the recent report that suggested a novel role for fetuin-A in the metastatic progression of prostate cancer, even though the mechanism involved was unclear [6]. 

To elucidate the potential role of fetuin-A in 3-D tumor cell growth, we employed the LNCaP prostate cancer cell line as our model system. We, therefore, routinely cultured the cells on low or ultra-low attachment plates to promote their growth as 3-D spheroids [14]. Armed with the knowledge that the growth medium supplemented with 10% fetal bovine serum (CM) contain relatively high concentrations of bovine serum exosomes and fetuin-A (~2 mg/mL), we questioned the role of these serum components in mediating 3-D growth. Interestingly, serum exosomes had previously been shown to promote anchorage-independent (3-D) growth of tumor cells in soft agar or Matrigel [16]. The data clearly demonstrated that bovine serum exosomes had no impact on the growth of LNCaP cells as spheroids since exosome-depleted complete medium supported robust 3-D growth. Removal of fetuin-A from exosome-depleted CM using hydroxyapatite nanoparticles, on the other hand, attenuated the growth of LNCaP cells as spheroids.

We demonstrated that the presence of fetuin-A was particularly required for the mediation of 3-D growth by extracellular vesicles, including exosomes, and small and large extracellular vesicles emanating from the LNCaP cells. Extracellular vesicles that were isolated in the absence of fetuin-A and incubated with LNCaP cells in the presence of BSA only failed to promote the 3-D growth of these cells. Meanwhile, those prepared or incubated with cells in the presence of fetuin-A dramatically influenced the growth of the tumor cells. Since the endogenous exosomes in complete medium derived from FBS played no role in the 3-D growth of LNCaP cells, it means that over time, fetuin-A immobilized exosomes and other extracellular vesicles secreted from the cells in an autocrine or paracrine fashion to the cell surfaces of recipient cells to promote 3-D growth. It is these tumor extracellular vesicles that have 3-D growth signaling potential. It has been reported that these extracellular vesicles have the potential to transmit growth signaling not only in tumor cells but also in normal cells associated with the growing tumor [11,23].

It has been difficult to pinpoint the exact mechanism by which fetuin-A modulates tumor growth due to its ability to interact with a number of proteins [9,24], and yet this property is what could explain the numerous physiological roles that have been assigned to it [25]. Whereas in attached and spread cells, fetuin-A mediates the rapid endocytic uptake of extracellular vesicles, promoting adhesion, spreading, motility, and invasion [1,10], it ends up sequestered in intracellular compartments and hardly remains on the cell surface. For the first time, the present studies showed that fetuin-A remains on the cell surface in detached cells or cells growing as spheroids. Whereas the growth of LNCaP cells as spheroids in low attachment plates is perhaps the simplest example of the 3-D growth of tumor cells in vivo, the model system has limitations. The chief among these is the lack of supporting stromal cells and extracellular matrix components such as those in Matrigel. However, the nature of the interaction of exosomes and other extracellular vesicles with the more rounded cells that form the spheroidal colonies is significant and impactful in our quest to design the next generation of anti-tumor and anti-metastatic drugs.

Based on the data presented, the presence of fetuin-A on the cell surfaces of detached cells suggests that it is a scaffolding protein or part of the cell surface scaffolding complex that secures signaling platforms such as extracellular vesicles on the surfaces of non-adhered recipient cells. Indeed the arrangement of protein domains on the cell surface of adhered and spread cells is complex. Detachment resulting in rounded cells may result in even more complex surface domains. These ideas have been advanced in the literature and represent the next frontier of cell signaling [26,27]. A case in point is the ability of fetuin-A to sequester EGF-loaded LEV on the surfaces of spheroid cells. This type of signaling mechanism, dubbed ‘rececrine’, where receptors such as EGFR are incorporated into extracellular vesicles and then transferred to recipient cells to mediate growth signaling, has recently been proposed [28]. We have thus provided direct experimental evidence supporting this modality of information exchange. It appears to be a very efficient way of directing growth signals to the rapidly expanding cancer cells.

In summary, we have demonstrated the functional requirement for fetuin-A in the mediation of 3-D or spheroid growth of LNCaP prostate cancer cells. Whereas fetuin-A is internalized to promote growth, motility, and invasion in the tumor cells growing in 2-D, during 3-D growth, fetuin-A remains on the cell surfaces, where it acts as a scaffolding protein sequestering extracellular vesicles emanating from the tumor cells. The vesicles immobilized on the cell surface are able to transmit growth signals such as MAP kinase signaling cascade to promote 3-D growth. Taken together, the data suggest that fetuin-A participates not only in the motility and invasion of the prostate cancer cells but it also plays a crucial role as a scaffolding protein in the growth of primary tumor cells as well as colonization and expansion of the cells in the distant organs such as bone [6]. Nevertheless, more prostate cancer cell lines and tissues need to be analyzed in order to generalize the role of fetuin-A in prostate cancer progression.

## 4. Materials and Methods

### 4.1. Cells

LNCaP, a prostate cancer cell line, was purchased from ATCC, Manssas, VA, USA. The line was maintained in RPMI-1640 medium supplemented with 10% FBS and 1X antibiotic/antimycotic (50:50) (Life Technologies, Grand Island, NY, USA). The cells were maintained in a humidified CO_2_ incubator at 37 °C. The serum-free medium (SFM) used in the studies contained 1% (*w*/*v*) bovine serum albumin. 

### 4.2. Materials

For 2-D cultures, the cells were cultured in CellSTAR Tissue culture treated 75 cm^2^ flasks (Greiner Bio-one, Monroe, NC, USA), and experiments were conducted using tissue culture treated 96-well, flat-bottom Falcon plates. For 3-D cultures, the cells were maintained in Corning 75 cm^2^ ultra-low attachment flasks, and most of the experiments were performed using Polystyrene Non-Treated 96-well low attachment plates (CellTreat, Pepperell, MA, USA) or ultra-low attachment Costar 24-well, flat-bottom culture plates (Corning, Durham, NC, USA). Rhodamine isothiocyanate labeled EGF was purchased from ThermoFisher, Waltham, MA, USA. Fetuin-A was purchased from Sigma (Sigma, St Louis, MO, USA) and further purified as described [9].

### 4.3. Isolation of Extracellular Vesicles

The LNCaP cells were detached either with 2 mM EDTA or trypsin/EDTA and quenched with complete medium. The detached cells were pelleted and re-suspended in 15 mL centrifuge tubes in either SFM (containing 1% BSA) or SFM containing fetuin-A (2 mg/mL) or complete medium (~1 × 10^9^ cells/tube). The cells were incubated at 37 °C with rotation for 45 min. At the end of incubation, the cells were pelleted by centrifugation (700× *g*) for 5 min and the supernatant centrifuged at 1500× *g* for 10 min to pellet dead cells and other cellular debris. The resulting supernatant was centrifuged at 3000× *g* for 15 min to pellet large extracellular vesicles (LEV). The resulting supernatant was centrifuged at 21,000× *g* for 30 min to pellet small extracellular vesicles (sEV). The resulting supernatant was centrifuged at 100,000× *g* for 2 h to pellet exosomes. The dimensions of the vesicles were determined by a nanoparticle analyzer (ZetaView Z-NTA Particle Matrix, Mebane, NC, USA). For each measurement, two cycles were performed by scanning 11 cell positions each and capturing 30 frames per position. Polystyrene beads 100 nm in size (NIST traceable standard) were used for the focus and calibration of the instrument. 

### 4.4. Three-Dimensional Growth Assays (3-D)

The cells were dislodged with EDTA or Trypsin/EDTA as described above. The cells were then plated as a single-cell suspension in ultra-low attachment 24-wells (1 × 10^4^ cells/well) or low attachment 96-well plates (5000 cells/well) in either SFM, fetuin-A (2 mg/mL), or complete medium for up to 10 days at 37 °C in a humidified CO_2_ incubator. The number of spheroidal colonies (Mean ± SD) of different sizes within 20× microscopic fields (N = 6) of cells growing under different conditions was determined. Complete medium was depleted of fetal bovine exosomes by subjecting it to 100,000× *g* ultra-centrifugation for 2 h. Part of the resulting exosome-free complete medium was incubated with hydroxyapatite nanoparticles (HA) (Sigma, St Louis, MO, USA) (50 mg HA/10 mL of medium) at 4 °C for 24 h to remove fetuin-A (CM fetuin-A depleted). Medium containing 10% adult human serum was also incubated with hydroxyapatite nanoparticles to remove fetuin-A as above. After 24 h, the hydroxyapatite particles were removed by centrifugation (5000× *g*), and the supernatant filter was sterilized and used in the experiments.

### 4.5. Labeling of Proteins and Extracellular Vesicles with Rhodamine Isothiocyanate Isothiocyanate

Fetuin-A, rabbit polyclonal antibodies against fetuin-A (Sigma, Saint Louis, MO, USA), as well as large extracellular vesicles (LEV), were labeled with rhodamine isothiocyanate. Briefly, 1 mg of purified fetuin-A, anti-fetuin-A antibody, and 1 mg of LEV at a concentration of 5 mg/mL in 0.1 M sodium bicarbonate (pH 9) was incubated with 50 µg of rhodamine isothiocyanate (1 mg/mL) in sterile DMSO at 4 °C for 8 h or overnight. The reaction was stopped by adding NH4Cl (50 µM) and incubated for another 2 h at 4 °C. The labeled proteins and vesicles were separated from unreacted label by passing twice through desalting columns (HiTratp^TM^, GE Healthcare, Chicago, IL, USA). The pass-through LEV was further pelleted at 3000× *g* for 15 min and then resuspended in SFM at 1 mg/mL. Labeled fetuin-A and the polyclonal antibodies were concentrated in 10,000 Mwt cut-off filters and then reconstituted to 1 mg/mL in SFM.

### 4.6. Cell Surface vs. Intracellular Disposition of Labeled Large Extracellular Vesicles

It has been demonstrated that large extracellular vesicles, also known as oncosomes, promote 3-D or anchorage-independent growth of tumor cells [29,30,31]. It is generally assumed that they do so by transmitting growth signals from one tumor cell to another in an either autocrine or paracrine fashion. Such mechanisms could involve the interaction of growth factors such as EGF and their receptors on LEV or, alternatively, the endocytic uptake of the vesicles, which then transfer oncogenic proteins, miRNA, or lncRNA to recipient cells, as has been demonstrated for exosomes [30]. Here, we questioned whether labeled LEV would remain on the cell surface or be internalized, in which case, the labeled vesicles would be metabolized or degraded in the endosomal compartments, and the fluorescence associated with them would decrease over time. For this reason, we added rhodamine isothiocyanate-labeled LEV (15 µg/well) to LNCaP cells growing in the presence of either BSA or fetuin-A (2 mg/mL) in low- or high-attachment plates 96-well plates. We then followed their growth over a 5-day period, with images acquired by a Keyence BZ-X800 digital Fluorescence Microscope (Keyence, Elmwood Park, NJ, USA).

### 4.7. Cell Surface vs. Intracellular Disposition of Labeled EGF Associated with LEV

It has been proposed that a plausible mechanism by which EGF promotes 3-D growth is to be associated with LEV, such as oncosomes loaded with EGFR [32,33,34]. The vesicles containing the EGF receptor could remain on the cell surface or be internalized for this to happen. We, therefore, incubated 4 µg of rhodamine isothiocyanate-labeled EGF (ThermoFisher, Waltham, MA, USA) with 1 mL of LEV (1 mg/mL) isolated from LNCaP in the presence of fetuin-A, for 30 min at 37 °C. After centrifugation of the incubation mixture at 3000× *g* for 15 min, the resulting pellet was reconstituted in SFM (~1 mg/mL) and added (15 µg/well) to LNCaP cells (5000 cells/well) seeded in low- or high-attachment 96-well plates in the presence of fetuin-A.

### 4.8. Cell Surface vs. Intracellular Disposition of Fetuin-A

We previously demonstrated that fetuin-A does not remain on the cell surface in attached and spread cells (2-dimensional growth). It is quickly endocytosed, and within a 2 h period, it is mainly confined to intracellular compartments [10]. We, therefore, repeated these experiments to determine whether the same process occurred in detached and spherical cells. To do this, the LNCaP cells were allowed to grow in the presence of fetuin-A (2 mg/mL), either in tissue-culture treated 96-well (cell attachment and spreading) or in untreated 96-well plates (low attachment plates). Labeled fetuin-A (rhodamine isothiocyanate) was added to the cells on high-attachment or low-attachment wells and monitored over a 72–96 h period. Images were acquired by a Keyence epifluorescence microscope.

#### 4.8.1. Cell Surface Fetuin-A on Attached and Spread Cells

We also employed a different strategy to observe the cell surface fetuin-A. LNCaP cells were allowed to attach and spread for 48 h on glass coverslips in the presence of fetuin-A (2 mg/mL). The cells were washed (3×) in SFM and then fixed in 3.5% paraformaldehyde. They were washed once more and incubated with rhodamine isothiocyanate-labeled rabbit anti-fetuin-A (1:100 dilution) for 1 h at room temperature. The cells were then washed 5×, and the coverslips were turned upside down on a drop of anti-fade mounting solution containing DAPI on Microscopy slides. The slides were then observed under an A1R confocal microscope. 

#### 4.8.2. Intracellular Fetuin-A in Attached and Spread Cells

To observe intracellular fetuin-A, LNCaP cells were once more allowed to attach and spread for 48 h on glass coverslips in the presence of fetuin-A (2 mg/mL). The cells were washed 3× in SFM and then fixed for 5 min in cold methanol. The cells were washed once more in SFM and then incubated with rhodamine isothiocyanate-labeled anti-fetuin-A for 1 h at room temperature. The coverslips were then processed as described above and observed under a confocal microscope. 

#### 4.8.3. Cell Surface Expression of Fetuin-A on Detached Spherical Cells

In order to determine whether fetuin-A remains on the cell surface or is internalized in rounded cells, we incubated detached LNCaP cells with purified fetuin-A (2 mg/mL) for 30 min at 37 °C with end-on-end rotation. The cells were then washed 5× with cold FACS buffer (PBS containing 1% BSA and 0.1% sodium azide). The cells were fixed in cold PBS containing 3.5% paraformaldehyde for 15 min and washed once again with cold FACS buffer. The fixed cells were incubated in cold FACS buffer containing rabbit non-immune IgG (1:100 dilution) (unlabeled control) or FACS buffer containing rhodamine isothiocyanate-labeled rabbit anti-fetuin-A (1:100 dilution) or FACS buffer containing rhodamine isothiocyanate-labeled rabbit anti-fetuin-A (1:100 dilution) and excess unlabeled rabbit anti-fetuin-A (1:5 dilution) in Eppendorf tubes with rotation for 1 h at room temperature. The cells were washed 5× with FACS buffer and then analyzed by a Cell Stream Flow Cytometer (Luminex, Austin, TX, USA). Initial gating to identify single cells was set using FSC Area and FSC Aspect Ratio parameters. Rhodamine median fluorescence intensity was analyzed in the single-cell populations using zone D and channel 4 of the CellStream for an excitation wavelength of rhodamine B isothiocyanate at 544 nm and emission wavelength of 576 nm. The data were analyzed by FlowJo software (TreeStar, San Carlos, CA, USA).

### 4.9. Western Blot Analysis

We sort to determine the role of fetuin-A in the activation of AKT and MAP kinase signaling pathways. Briefly, LNCaP cells were grown in 6 or 8 tissue cultures treated with high attachment until ~70% confluent. The medium was replaced with SFM, and the cells serum-starved for 48 h. The cells in some of the flasks were detached with 2 mM EDTA and then transferred to new ultra-low attachment flasks (3-D) in SFM containing BSA (2 mg/mL) or fetuin-A (2 mg/mL) or 10% FBS or 10% FBS depleted (minus) of fetuin-A and incubated at 37 °C for 2 h or 24 h. In the other flasks (2-D) after serum starvation, the SFM was replaced with SFM containing BSA (2 mg/mL) or fetuin-A (2 mg/mL) or 10% FBS and incubated at 37 °C for 2 h or 24 h. At the end of each incubation period, whole-cell extracts from LNCaP cells were prepared in a radio-immunoprecipitation assay (RIPA) buffer (50 mM Tris/HCl, pH 7.4, 1% NP-40, 0.1% sodium deoxycholate, 150 mM NaCl, 1 mM EDTA, and freshly added protease and phosphatase inhibitor cocktail). Thirty micrograms of protein from each flask was loaded to 4–12% SDS/PAGE. The protein bands were transferred to polyvinylidene fluoride membranes. After blocking with 5% nonfat milk, the membranes were incubated with antibodies to phospho-AKT, total AKT, phospho-ERK, total ERK, and β-actin (Santa Cruz, Dallas, TX, USA) overnight at 4 °C. After incubation with the corresponding secondary antibodies for signal detection, the membranes were exposed to chemiluminescence (Perkin Elmer, Waltham, MA, USA).

### 4.10. Experimental Rigor and Statistics

Each of the Figures is representative of at least two separate experiments. In Figure 1 and Figure 2, we counted the numbers (mean ± SD) of small-sized (<8 cells), medium-sized (>8 cells but less than 30 cells), and large-sized (>30 cells) spheroids in a 20× microscopic field of view. The presence of medium-sized and/or large-sized spheroid colonies, regardless of the numbers, indicated 3-D growth. The presence of only small spheroid colonies (<8 cells) indicated a lack of 3-D growth.
